# Ergot Alkaloids at Doses Close to EU Regulatory Limits Induce Alterations of the Liver and Intestine

**DOI:** 10.3390/toxins10050183

**Published:** 2018-05-01

**Authors:** Viviane Mayumi Maruo, Ana Paula Bracarense, Jean-Paul Metayer, Maria Vilarino, Isabelle P. Oswald, Philippe Pinton

**Affiliations:** 1 Universidade Federal do Tocantins, Araguaína 77824-838, Brazil; vmmaruo@hotmail.com; 2 Laboratory of Animal Pathology, Universidade Estadual de Londrina, Londrina 86057-970, Brazil; ana.bracarense29@gmail.com; 3 ARVALIS-Institut du Végétal, Station expérimentale, 41100 Villerable, France; jp.mj.metayer@free.fr (J.-P.M.); M.Vilarino@arvalis.fr (M.V.); 4 Toxalim (Research Centre in Food Toxicology), Université de Toulouse, INRA, ENVT, INP-Purpan, UPS, 31027 Toulouse, France; philippe.pinton@inra.fr

**Keywords:** *Claviceps*, liver, digestive tract, mycotoxin, sclerotia, ergot alkaloids, toxicity

## Abstract

An increase in the occurrence of ergot alkaloids (EAs) contamination has been observed in North America and Europe in recent years. These toxins are well known for their effects on the circulatory and nervous systems. The aim of this study was to investigate the effect of EAs on the liver and on the intestine using the pig both as a target species and as a non-rodent model for human. Three groups of 24 weaned piglets were exposed for 28 days to control feed or feed contaminated with 1.2 or 2.5 g of sclerotia/kg, i.e., at doses close to EU regulatory limits. Contaminated diets significantly reduced feed intake and consequently growth performance. In the liver, alteration of the tissue, including development of inflammatory infiltrates, vacuolization, apoptosis and necrosis of hepatocytes as well as presence of enlarged hepatocytes (megalocytes) were observed. In the jejunum, EAs reduced villi height and increased damage to the epithelium, reduced the number of mucus-producing cells and upregulated mRNA coding for different tight junction proteins such as claudins 3 and 4. In conclusion, in term of animal health, our data indicate that feed contaminated at the regulatory limits induces lesions in liver and intestine suggesting that this limit should be lowered for pigs. In term of human health, we establish a lowest observed adverse effect level (LOAEL) of 100 μg/kg body weight (bw) per day, lower than the benchmark dose limit (BMDL) retained by European Food Safety Authority (EFSA) to set the tolerable daily intake, suggesting also that regulatory limit should be revised.

## 1. Introduction

Ergot is a parasitic fungus that belongs to the *Claviceps* genus. It forms on various grains and grasses a dark mass of mycelium called sclerotia producing toxic secondary metabolites, the ergot alkaloids (EAs). These mycotoxins gained notoriety in the Middle Ages because of mass poisonings in Europe, characterized by gangrene and convulsions [1].

More than 50 different EAs have been identified so far. The main EAs produced by *Claviceps* species are ergometrine, ergotamine, ergosine, ergocristine, ergocryptine and ergocornine. Their toxicity is linked to their structural similarity with dopamine, noradrenaline, adrenaline and serotonin, enabling binding to the biogenic amine receptor and the interruption of neurotransmission [2]. Typical clinical symptoms of ergot poisoning are vasoconstriction, which may progress into gangrene, disruption of reproduction, abortion, neurotoxic signs including feed refusal, dizziness and convulsions, agalactia and adverse effects to the cardiovascular system [1,3,4,5]. Although acute poising has become rare, EAs are still a source of concern because they continue to be detected in cereals and cereal products in Europe and North America [6,7]. Moreover, the occurrence of *Claviceps purpurea* (*C. purpurea*) infections has been increasing in the last few years [8,9,10,11].

Currently, regulations are based on the quantities of ergot sclerotia. Codex Alimentarius established maximum levels for sclerotia of *C. purpurea* in wheat and durum wheat intended for processing for human consumption at 0.5 g/kg and 5 g/kg, respectively [12]. In animal feed, the European Commission fixed the maximum content at 1 g/kg of feed stuff containing unground cereals [13]. Recently, the EFSA Panel on Contaminants in the Food Chain conducted a risk assessment and proposed a tolerable daily intake (TDI) of 0.6 μg total EA/kg body weight (bw) per day [1] for humans.

The aim of this study was to investigate the effects of ingestion of EA-contaminated feed at concentrations close to the regulatory limits. We chose to perform the experiment in pigs as they may be exposed to EAs. In addition, they represent a choice species as a biomedical model for human toxicology, due to their similarities in anatomy, genetics and pathophysiology [14].

We mainly focused on the effects of EAs on the intestine and the liver. The intestine is of particular importance since it represents the first barrier against food contaminants and foreign antigens. The jejunum is one of the major intestinal sites of absorption, including the absorption of toxins. Intestinal epithelium integrity plays a critical role in the maintenance of the physical and immune barrier that is achieved by an ensemble of well-organized structural and secretory components including tight junctions, mucus and cytokines [15]. Most of the metabolization processes for xenobiotics, including mycotoxins, occur in the liver [16]. The metabolizing enzymes of the cytochrome P450 family are involved in and induced by ergot metabolism [17]. Moreover, certain hepatic enzymatic activities were found to be inhibited by ergotamine and ergometrine [18]. Reports in the literature on the effects of ergot on the intestine and the liver are rare, especially concerning biochemical and morphological parameters.

## 2. Results

### 2.1. Effects of Ergot Alkaloids on Clinical Signs, Growth and Feed Intake

The effects of ergot were first investigated in pigs exposed for 28 days at doses of 1.2 and 2.5 g sclerotia/kg feed, which are just above the European regulatory limit. When assessing clinical signs, no typical symptoms of acute toxicity, such as convulsions or muscle spasms, or necrosis of the extremities were observed. Similarly, no vomiting or fever was observed in animals exposed to ergot.

**Figure 1:**
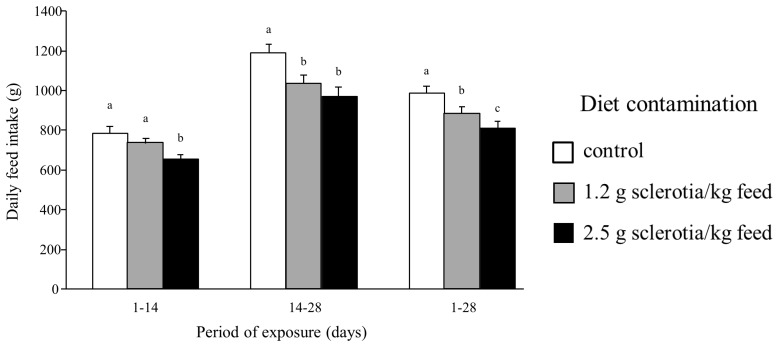
Daily feed intake of piglets fed ergot sclerotia for 28 days (*n* = 24/group). Values are means with standard errors of the mean represented by vertical bars. ^a, b, c^ mean values with different letters are statistically different (*p* < 0.05).

The effects of ergot on feed intake were already detected the second week in animal exposed to the highest dose. For animals exposed to the lower dose of ergot, this reduction only appeared in the last 14 days of treatment (Figure 1). During the experimental period, the daily feed intake of animals exposed to the higher dose of ergot was reduced by about 18% in comparison with control group (Figure 1). Taking into account the whole period of treatment, the daily feed intake was significantly reduced as a function of ergot content (Figure 1).

The reduction in feed ingestion led to a decrease of animal weight gain that was significant in the group exposed to the higher dose of EA (data not shown).

### 2.2. Effects of Ergot Alkaloids on Hematology and Blood Chemistry

Hematology and serum biochemical analysis were performed at the end of the experiment. When animals were exposed to ergot, the percentage of neutrophils was reduced and the decrease was significant in the group exposed to the lower dose (Table 1). The percentage of lymphocytes also increased in animals exposed to ergot, with a significant difference in animals exposed to the higher dose (Table 1).

Biochemical analysis revealed a significant dose-dependent reduction in the levels of creatine kinase in animals exposed to ergot. In addition, the level of cholesterol decreased and that of glucose increased upon exposure to ergot (Table 2).

### 2.3. Alterations of Tissue Morphology Caused by Ergot Alkaloids

**Figure 2:**
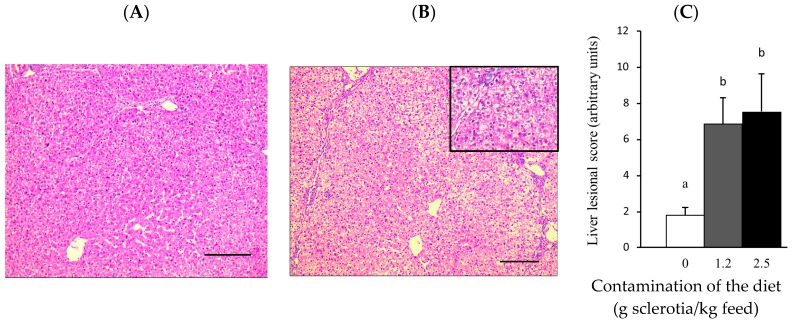
Liver of piglets fed ergot diets. (**A**) Control piglet. Normal liver. HE. Objective 10×; (**B**) Piglet fed 2.5 g ergot sclerotia/kg feed. Disorganization of hepatic cords and periportal hepatocyte vacuolation. HE. Objective 10×. Insert: Hepatocyte vacuolation. HE. Objective 40×; (**C**) Liver lesion score. Values are means with their standard errors of mean represented by vertical bars (*n* = 6 animals). Mean values with different letters are significantly different (*p* < 0.05). Bar = 100 μm.

**Figure 3:**
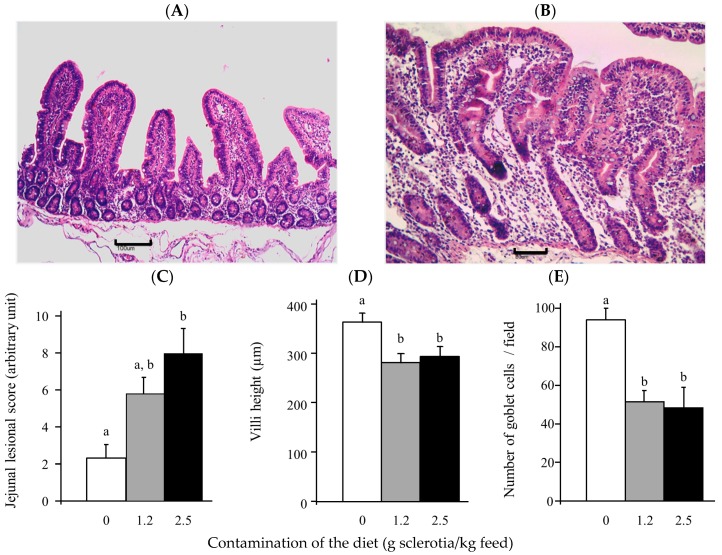
Effect of ergot exposure on jejunum of pigs receiving a control diet or a diet contaminated with 1.2 or 2.5 g ergot sclerotia/kg feed for 28 days. Jejunal section with hematoxylin-eosin (HE), Objective 10×: (**A**) Control piglet; (**B**) Piglet fed 2.5 g ergot sclerotia/kg feed; (**C**) Lesion score according to the occurrence and severity of lesions observed on formalin-fixed tissue sections stained with HE; (**D**) Villi height measured on formalin-fixed tissue sections stained with HE; (**E**) Number of goblet cells observed on formalin-fixed tissue sections stained with Schiff’s periodic acid. Values are means of the score, villi height and number of goblet cells/field respectively, with standard errors of the mean represented by vertical bars (*n* = 6 animals). ^a,b^ mean values with different letters are statistically different (*p* < 0.05).

The effects of ergot on the liver and the intestine were investigated through histological analyses. Exposure to ergot led to mild to moderate lesions of the liver (Figure 2) and the jejunum (Figure 3) of pigs.

In the liver, tissue disorganization of hepatic cords, inflammation and vacuolation of hepatocytes, megalocytosis and necrosis were the main morphological alterations (Figure 2B). Furthermore, animals fed the contaminated diets presented a significant increase in the lesion liver score (Figure 2C).

The main histological changes observed in the jejunum were villi atrophy, edema of lamina propria and cytoplasmic vacuolation of enterocytes. As shown in Figure 3C, animals exposed to the higher dose of ergot displayed a significant increase of the lesion score in the jejunum compared to control animals (3.4 fold increase, *p* < 0.005). Morphometrical analysis revealed a significant decrease in villi height (1.2 fold decrease at both doses) (Figure 3D). The number of goblet cells decreased significantly in the jejunum (mean 1.6 fold decrease, *p* < 0. 001) of piglets exposed to ergot (Figure 3E). Similar effects were observed in jejunum areas with Peyer’s patches (data not shown).

### 2.4. Effects of Ergot Alkaloids on mRNA Expression in the Jejunum

**Figure 4:**
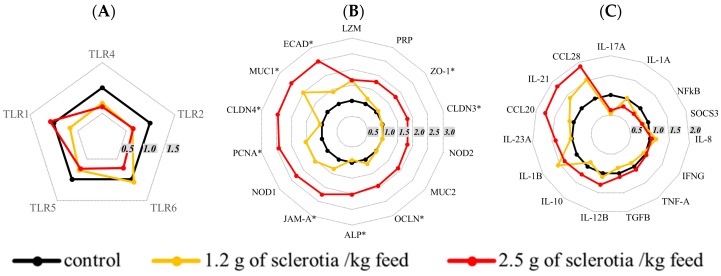
Expression of selected genes in the jejunum of pigs fed with a control diet or a diet including ergot alkaloids for 28 days. mRNA levels were measured by RT-qPCR. The three panels present different groups: TLR-encoding genes (**A**), junctional protein-encoding genes (**B**) and immune mediator-encoding genes (**C**). The gene expression levels for the control group are shown in black (mean value adjusted to 1 for this group) and those for the groups exposed to 1.2 and 2.5 g sclerotia/kg feed ergot are shown in orange and red, respectively (*n* = 6 animals/group). * statistical difference in gene expression between control animals and pigs exposed to the highest dose of ergot (*p* < 0.05).

Given the lesions induced by ergot exposure in the jejunum and due to the lack of studies on the toxicity of EAs in this organ, the expression of 34 genes coding for junctional proteins, inflammatory and immunological mediators was evaluated by real-time-quantitative PCR (RT-qPCR) in the jejunum. The expression profile of mRNA expression of different toll-like receptors (TLR) and cytokines (TLR4, nuclear factor kappa B (NFkB), interleukin (IL)-6, IL-8, tumor necrosis factor-α (TNFA)) showed a tendency to downregulation. In animals exposed to the higher dose of ergot, analysis of mRNA expression of junctional proteins revealed a significant increase in claudin-3 (CLDN3), claudin-4 (CLDN4), occludin (OCLN), zonula occludens-1 (ZO-1), junctional adhesion molecule (JAM-A) and E-cadherin (ECAD) (Figure 4). The expression of genes coding for mucin-1 (MUC1), alkaline phosphatase (ALP) and proliferating cell nuclear antigen (PCNA) was also significantly increased. Altogether, the overexpression of the genes involved in maintaining the structure of the intestinal mucosa may reflect an attempt by the tissue to reestablish its function.

## 3. Discussion

The aim of this study was to investigate the effects of ingestion of EA-contaminated feed on the liver and intestine in pig. The levels of sclerotia in the feed were 1.2 g sclerotia or 2.4 mg total ergot alkaloids/kg. Using these concentrations that were close to the regulatory limits for 28 days, we observed not only effects on animal performances but also lesions in the liver and the intestine. This study shows that the regulation of 1 g sclerotia/kg of feed materials is probably not protective enough for pigs and deserves to be re-evaluated. In terms of human health, a LOAEL of 100 μg/kg bw per day could be established from the present data. This is lower than the BMDL associated with a 10% response of 330 μg/kg bw per day, which is set by EFSA as the tolerable daily intake for human. The present experiment thus provides evidence of the necessity to re-examine the current TDI for humans.

Considering the mean feed intake of these animals (1163 and 962 g/day after respectively, two and four weeks of exposure) and their mean weight (17.5 and 27.8 kg after respectively, two and four weeks of exposure), it represents an exposure to ergot alkaloids of 159 and 83 μg/kg bw per day after two and four weeks of exposure, respectively. The lowest observed adverse effect level (LOAEL) is lower than the benchmark dose limit (BMDL) associated with a 10% response of 330 μg/kg bw per day retained by EFSA to set the tolerable daily intake (TDI) of 0.6 μg/kg bw per day [1], based on the vasoconstrictive effects observed in a rat model exposed to ergotamine for 13 weeks [19]. Given the interspecies differences in the observed effects, pig could be considered as a relevant species for ergot alkaloids risk assessments. These data reinforce the need to increase the knowledge on the effects of EAs and suggest a revision of the TDI for EAs.

As already reported in other studies, we observed that the ingestion of EAs damages the zootechnical parameters as measured by feed intake and average daily gain [17,20]. Reduction of weight gain appears to be caused by the reduction in feed intake, which in turn may be explained by the action of the EAs on serotoninergic receptors in the gut, thereby affecting motility [2]. The composition and proportion of EAs in sclerotia can vary but the total EA content appears to be more important than the composition of EA for the alteration of animal performances [21].

The consumption of ergot also affected intestinal integrity, as revealed by histological and molecular analyses, and might have contributed to growth impairment of the animals. Histological and morphometric evaluation of the intestinal tissue revealed a sensitive endpoint for the evaluation of the effects of mycotoxin exposure. Modifications in villi height reflect changes in the balance between enterocyte proliferation and apoptosis. The increase in the lesion score and villi flattening in the intestine of animals exposed to EAs could be explained by their action on serotoninergic receptors present in epithelial intestinal cells, primary afferent neurons and secretor motor neurons of the intestine [22]. In addition to the action of EAs on the serotoninergic system, recent in vitro studies demonstrated that EAs also cause cytotoxicity and apoptosis in intestinal HT-29 and liver HepG2 cell lines, providing evidence for an intricate mode of action [23]. Additionally, the number of goblet cells was reduced in animals exposed to EAs, revealing impairment in the intestinal barrier function [15,24]. Taken together, these alterations could lead to a compensatory upregulation of mRNA levels of CLDN3, CLDN4, OCLN, ZO-1, JAM-A, ECAD, ALP and PCNA, as already described in the intestine of mice [25] or in intestinal epithelial cells exposed to deoxynivalenol [26]. Immunoregulatory mechanisms in the intestine play a crucial role in the defense of the organism [27]. The pattern recognition receptors (PRR) are membrane-bound receptors that specifically bind to pathogen-associated molecular patterns (PAMPs) shared by various microorganisms. PRR are highly expressed in the jejunum and ileum of pigs and trigger an inflammatory response, involving the activation of myeloid differentiation 88 and NFkB [28]. In the present work, mRNA expression of TLR-1 2, 4, 5 and 6 as well as TNF-A, IL-1A, IL-8 and IFNG tended to be downregulated in the jejunum of animals exposed to EAs. This could reduce tolerance to commensal microbiota and the organism’s ability to trigger inflammation response against pathogens, thereby increasing susceptibility to infections [29].

Besides the action of EAs on the intestine, their effects were also studied on the liver where tissue disorganization, inflammation and necrosis were observed and assessed by a significant increase of the lesion liver score. We observed that the consumption of EA-contaminated feed affected the levels of cholesterol, glucose and creatine kinase, parameters correlated with adiposity and muscle growth [30]. Hypocholesterolemia could correlate with the malabsorption of nutrients as well as with an altered lipid metabolism that often accompanies hepatic disease [31]. Glucose levels were elevated in the group that received the higher ergot content. In the post-absorptive phase, glucose is transported from its site of uptake in the gut to the sites of glycogen storage, principally the liver and muscles [32]. The increase in glucose level indicates glycogen mobilization from the storage sites to provide energy to the organism. In the present study, the morphological changes observed in hepatocytes could be associated with functional alterations such as lipid metabolism and glycogen storage. The reduction in creatine kinase may indicate hypoplasia, atrophy or the mild destruction of skeletal muscles [31,32]. EAs induce vasoconstriction through binding with serotoninergic receptors in the smooth muscle cells of vessels walls [5]. Therefore, an association between a relative ischemia and changes in creatine kinase could be hypothesized. In short, the biochemical alterations observed in animals exposed to ergot could be correlated to liver and mild muscle lesions [30].

## 4. Conclusions

The contamination of cereals by EAs has recently been shown to be increasing in different countries and is a source of concern for human and animal health. This study confirmed that EA reduces the performances of pigs, and demonstrated that EAs provoke hepatotoxicity, as revealed by deleterious effects on tissue morphology, and mild biochemical alterations. Moreover, EA alters the intestinal epithelium, reduces the number of goblet cells and modulates the expression of genes involved in immune response and barrier function. This suggests that ingestion of EA-contaminated feed may alter the intestinal barrier function, predisposing animals to enteric infections and potentially causing hepatotoxicity.

From the point of view of pig health, our data indicate that feed contaminated at the regulatory limits induces deleterious effects in the liver and intestine in pigs, suggesting that the regulatory limit is not adequately protective. From the point of view of human health, this study reveals the sensitivity of pigs to EAs with a LOAEL lower than the BMDL obtained in rats, which is employed by EFSA to set the tolerable daily intake for human. The present data should be considered in a further assessment of human risk of exposure to EAs.

## 5. Materials and Methods

### 5.1. Experimental Diets

Experimental diets, based on wheat, corn and soybean and formulated according to the CORPEN (1996) norms are detailed in Table 3. Two batches of sclerotia obtained from contaminated wheat were used to prepare the contaminated diets. Levels of sclerotia of 0 (control), 1.2 g/kg (dose 1) and 2.5 g/kg (dose 2) were used, the first dose being close to the regulatory limit of 1 g/kg [13].

### 5.2. Ergot Alkaloid Composition of the Diets

The quantities of EAs were determined by liquid chromatography coupled with a tandem mass spectrometry (LC/MS/MS) (TSQ Quantum Ultra, ThermoFisher Scientific, Villebon sur Yvette, France) at Qualtech laboratory (Vandœuvre, France).

After preparation, diets were analyzed four times and the mean EA concentrations were 2.36 and 5.05 mg/kg, for diets with ergot dose 1 and 2, respectively. The most abundant alkaloid was ergotamine, followed by ergosine, ergocristine and their corresponding-inine epimers (Table 4). The amount of the-ine isomers was approximately two-thirds of total alkaloids.

Contamination with other mycotoxins was investigated. Deoxynivalenol was naturally present in the wheat (19 μg/kg) and corn (171 μg/kg) but at an insignificant rate. Other mycotoxins, including DON acetylated, nivalenol, T2, HT-2, zearalenone and fumonisin were below the detection limit.

### 5.3. Animals

Animal experiments were carried out at ARVALIS—Institut du végétal facility (Villerable, France) in accordance with the guidelines for protection of animals used for scientific purposes issued by French Ministry of Higher Education and Research. Seventy-two castrated, 21 day-old crossbreed (P76 X Naïma) piglets (mean weight 10.7 ± 0.9 kg) were housed in the facility with free access to control starter feed and water. When they were 34 days old, they were housed in individual pens and assigned to three groups of 12 males and 12 females one group fed the control diet, another ergot dose 1 and the third ergot dose 2 diet. At the end of the experiment (62 days old) their mean weight was 27.9 ± 3.8 kg, 27.8 ± 2.6 kg, 25.6 ± 3.1 kg in the control group, ergot dose 1 and ergot dose 2 diet groups, respectively.

### 5.4. Experimental Setting and Sample Collection

Animals received experimental diets for a period of 28 days. Weight and consumption were measured on the first day of the experiment, 14 days later and on the last day of the experiment. The animals were observed daily to detect possible signs of ergot intoxication such as balance disorders or necrosis of the extremities.

At the end of the experiment, blood samples were taken from the external jugular vein of all the animals for biochemical and hematological analyses. In addition, six male animals in each group were euthanized and liver, jejunum and jejunum with Peyer’s patches were sampled in 10% buffered formalin (Sigma, Saint-Quentin Fallavier, France) for histopathological analysis or immediately quick-frozen in liquid nitrogen and stored at −80 °C until RNA extraction [33].

### 5.5. Hematology and Blood Chemistry

Hematological parameters (white and red blood cells, hemoglobin and platelets) were analyzed at Medibiolab (Vendome, France), with a Sysmex XT2000i automated hematology analyzer (Sysmex, Villepinte, France). Serum biochemistry was determined with a Pentra 400 Clinical Chemistry benchtop analyzer (Horiba, Les Ulis, France) at GenoToul-Anexplo platform (Toulouse, France).

### 5.6. Histomorphometrical Analysis

Formalin-fixed tissue samples were dehydrated in ethanol and embedded in paraffin wax. Sections (3 μm) were stained with hematoxylin-eosin (HE, Sigma) for histopathological analysis. A lesion score per animal was established by taking into account the severity of the lesion and its extent (intensity or observed frequency; scored from 0 to 3) as described previously [34]. Morphometry was evaluated in the jejunum by measuring the height of 30 randomly chosen villi using a MOTIC Image Plus 2.0 ML 1 image analysis system (Richmond, Canada, 2003), as described previously. In addition, Schiff’s periodic acid stain was used to evaluate goblet cell density (five fields/slide, objective 20×) [35].

### 5.7. Expression of mRNA by Real-Time PCR

Intestinal tissue was processed in lysing matrix D tubes (MP Biomedicals, Illkirch, France) containing Extract-All (Eurobio, Les Ulis, France) in a Fast-Prep FP120 instrument (MP Biomedicals) as described previously [36,37]. The concentrations and purity of RNA were determined using a Nanodrop nd1000 instrument (Labtech International, Paris, France). Total RNA samples were reverse-transcribed using a High Capacity cDNA-RT kit (Life Technologies, Saint Aubin, France). RT-qPCR was performed in 384-well plates in a ViiA 7 thermocycler (Life Technologies, Saint Aubin, France) as described previously [38]. All reactions were performed in duplicate and averages were used for further analysis. Primers for the real-time quantitative PCR are presented in Table 5. Among the different internal reference genes tested were Beta-2-microglobulin (B2M), peptidylprolyl isomerase A (PPIA—cyclophilin A) and ribosomal protein L32 (RPL32); the latter were selected for their stability of expression assessed with the NormFinder program [39]. Data from qPCR were analyzed with the LinRegPCR 2016. 2 program [40], enabling the determination of the starting concentrations (N0).

### 5.8. Statistical Analysis

The zootechnical parameters (daily feed intake and average weight gain) were measured on 24 piglets per group, and the differences in variance analysis were assessed with the Newman et Keuls post hoc test.

Histological data were measured on 6 animals per group and statistically analyzed by the free software Action 2.3 (Estatcamp, Campinas, SP, Brazil, 2003) using normality (Shapiro-Wilk’s test) and homogeneity (Bartlett) tests. When these two assumptions were met, the lesional score, the intestinal morphometry and the number of goblet cells were analyzed by ANOVA followed by Tukey’s test. For gene expression, quantification by qPCR and statistical analysis, the mRNA expression of target genes was normalized to the expressed housekeeping genes using REST© 2009 software (Qiagen, Valencia, CA, USA) which uses the pair-wise fixed reallocation randomization test as statistical model [44]. *P* values below 0.05 (*p* ≤ 0.05) were considered significant.

## Figures and Tables

**Table 1:** Hematological analysis of pigs fed 1.2 or 2.5 g ergot sclerotia/kg feed (*n* = 24/group).

Parameters	Contamination of the Diet (g of Sclerotia/kg Feed)		
	0	1.2	2.5
Red blood cells (T/L)	7.75 ± 0.12	7.80 ± 0.13	8.08 ± 0.13
Hemoglobin (g/dL)	10.45 ± 0.22 ^a,b^	10.09 ± 0.21 ^a^	10.97 ± 0.19 ^b^
Hematocrit (%)	36.6 ± 0.70 ^a,b^	35.52 ± 0.87 ^a^	38.31 ± 0.63 ^b^
Mean corpuscular volume (fL)	47.46 ± 0.93	45.67 ± 0.93	47.58 ± 0.91
White blood cells (10^3^/mm^3^)	14.5 ± 0.6	14.2 ± 0.9	16.5 ± 0.9
Neutrophils (%)	40.92 ± 2.51 ^a^	30.67± 1.84 ^b^	36.17 ± 2.55 ^a,b^
Eosinophils (%)	0.38 ± 0.15	0.54 ± 0.30	0.29 ± 0.14
Basophils (%)	0.08 ± 0.08	0.08 ± 0.08	0.00 ± 0.00
Lymphocytes (%)	53.96 ± 2.66 ^a^	63.96 ± 1.95 ^b^	58.79 ± 2.77 ^a,b^
Monocytes (%)	4.67 ± 0.42	4.75 ± 0.68	4.75 ± 0.63
Platelets/mm^3^	521.58 ± 32.67	465.00 ± 28.08	533.04 ± 32.39

^a, b^ mean values with different letters are statistically different (*p* < 0.05).

**Table 2:** Serum biochemical analysis of pigs fed 1.2 or 2.5 g ergot sclerotia/kg feed (*n* = 24/group).

Parameters	Contamination of the Diet (g of Sclerotia/kg Feed)		
	0	1.2	2.5
Alkaline phosphatase (U/L)	275.7 ± 24.5	237.6 ± 16.7	228.6 ± 16.1
Alanine aminotransferase (U/L)	37.3 ± 1.9	31.74 ± 1.5	33.32 ± 1.6
Amylase (U/L)	1783 ± 123	1815 ± 99	2074 ± 122
Aspartate aminotransferase (U/L)	56.0 ± 6.8	42.6 ± 5.4	44.4 ± 3.94
Creatine kinase (U/L)	3377 ± 396 ^a^	1924 ± 297 ^b^	1300 ± 252 ^b^
Lactate dehydrogenase (U/L)	962.8 ± 50.5	968.3 ± 57.7	1013.3 ± 72.5
Lipase (U/L)	6.45 ± 0.5	6.9 ± 0.3	5.9 ± 0.8
Albumin (μmol/L)	533.0 ± 9.7	530.4 ± 11.7	522.4 ± 13.3
T bilirubine (μmol/L)	11.2 ± 1.2	11.0 ± 1.2	7.4 ± 1.3
Cholesterol (mmol/L)	3.0 ± 0.1 ^a^	2.6 ± 0.1 ^b^	2.8 ± 0.1 ^a^
Creatinine (μmol/L)	61.9 ± 6.2	63.3 ± 6.5	71.7 ± 6.4
Glucose PAP (mmol/L)	4.4 ± 0.2 ^a^	4.7 ± 0.3 ^a^	5.4 ± 0.3 ^b^
Phosphorus (mmol/L)	3.1 ± 0.1	3.2 ± 0.1	3.0 ± 0.1
Total proteins (g/L)	63.5 ± 7.8	59.1 ± 7.4	62.5 ± 8.2
Urea (mmol/L)	4.7 ± 0.4	4.3 ± 0.3	3.5 ± 0.4

^a, b^ mean values with different letters are statistically different (*p* < 0.05).

**Table 3:** Chemical composition of the diets (% of dry matter DM).

Parameters	Contamination of the Diet (g of Sclerotia/kg Feed)		
	0	1.2	2.5
Total nitrogen	20.1	20.2	20.3
Raw cellulose	2.6	2.7	2.8
Lipids	4.3	4.4	4.4
Minerals	6.0	6.1	6.0

**Table 4:** Ergot alkaloid content in experimental feed (mg/kg).

Alkaloids (mg/kg Feed)	Contamination of the Diet (g of Sclerotia/kg Feed)	
	1.2	2.5
Ergotamine	0.52	1.03
Ergotaminine	0.24	0.58
Ergosine	0.29	0.58
Ergosinine	0.16	0.34
Ergocristine	0.26	0.47
Ergocristinine	0.18	0.40
Ergometrine	0.17	0.44
Ergometrinine	0.06	0.12
Ergocornine	0.15	0.30
Ergocorninine	0.11	0.29
Ergocryptine	0.13	0.30
Ergocryptinine	0.09	0.21
**Total alkaloids**	**2.4**	**5.1**

**Table 5:** Primer sequences of genes used for qRT-PCR analysis of the jejunum (F: forward; R: reverse).

Target Gene		Primer Sequence (5′–3′)	mRNA	Reference
Alkaline phosphatase (ALP)	F	AAGCTCCGTTTTTGGCCTG	ENSSSCT00000037252.1	[28]
	R	GGAGGTATATGGCTTGAGATCCA		
Beta-2-microglobulin (B2M)	F	TTCTACCTTCTGGTCCACACTGA	NM_213978.1	[41]
	R	TCATCCAACCCAGATGCA		
C-C Motif Chemokine Ligand 20 (CCL20)	F	GCTCCTGGCTGCTTTGATGTC	NM_001024589	Present study
	R	CATTGGCGAGCTGCTGTGTG		
C-C Motif Chemokine Ligand 28 (CCL28)	F	GGCTGCTGTCATCCTTCATGT	ENSSSCT00000018375	Present study
	R	TGAGGGCTGACACAGATTCTTCT		
Claudin 3 (CLDN3)	F	CTGCTCTGCTGCTCGTGCCC	AY625258.1	Present study
	R	TCATACGTAGTCCTTGCGGTCGTAG		
Claudin 4 (CLDN4)	F	CTGCTTTGCTGCAACTGCC	NM_001161637.1	[26]
	R	TCAACGGTAGCACCTTACACGTAGT		
E-cadherin (ECAD)	F	ACCACCGCCATCAGGACTC	NM_001163060.1	Present study
	R	TGGGAGCTGGGAAACGTG		
Interferon gamma (IFNG)	F	TGGTAGCTCTGGGAAACTGAATG	NM_213948	[28]
	R	GGCTTTGCGCTGGATCTG		
Interleukin 1A (IL-1A)	F	TCAGCCGCCCATCCA	NM_214029.1	[38]
	R	AGCCCCCGGTGCCATGT		
Interleukin 1B (IL-1B)	F	ATGCTGAAGGCTCTCCACCTC	NM_214055	[28]
	R	TTGTTGCTATCATCTCCTTGCAC		
Interleukin 8 (IL-8)	F	GCTCTCTGTGAGGCTGCAGTTC	NM_213867.1	[38]
	R	AAGGTGTGGAATGCGTATTTATGC		
Interleukin 10 (IL-10)	F	GGCCCAGTGAAGAGTTTCTTTC	NM_214041	[38]
	R	CAACAAGTCGCCCATCTGGT		
Interleukin 12B (IL-12B)	F	GGTTTCAGACCCGACGAACTCT	NM_214013.1	[38]
	R	CATATGGCCACAATGGGAGATG		
Interleukin 17A (IL-17A)	F	CCAGACGGCCCTCAGATTAC	NM_001005729.1	[38]
	R	GGTCCTCGTTGCGTTGGA		
Interleukin 21 (IL-21)	F	GGCACAGTGGCCCATAAATC	NM_214415	Present study
	R	GCAGCAATTCAGGGTCCAAG		
Interleukin 23A (IL-23A)	F	TTCTCTACACCCTGATGGCTCTG	ENSSSCT00000047550.1	Present study
	R	TCGGGCTGCAAGAGTTGC		
Junctional adhesion molecule A (JAM-A)	F	CGTGCCTTCATCAACTCTTCCTAT	NM_001128444.1	Present study
	R	CACAAGTGTAATCTCCAGCATCAGA		
Lysozyme (LZM)	F	GGTCTATGATCGGTGCGAGTTC	NM_214392.2	[28]
	R	TCCATGCCAGACTTTTTCAGAAT		
Mucin 1 (MUC1)	F	GCATTACAAACCTCCAGTTTACCT	AY243508.1	[42]
	R	CCCAGAAGCCCGTCTTCTTT		
Mucin 2 (MUC2)	F	GCAGCCTGTGCGAGGAA	XM_003122394.1	[42]
	R	TGTCATCATACACAGTGCCTTCTG		
Occludin (OCLN)	F	AGCTGGAGGAAGACTGGATCAG	U79554.1	[43]
	R	TGCAGGCCACTGTCAAAATT		
Nuclear Factor Kappa B (NFkB)	F	CCTCCACAAGGCAGCAAATAG	ENSSSCT00000033438	Present study
	R	TCCACACCGCTGTCACAGA		
Nuclear oligomerization domain 1 (NOD1)	F	TGGGCTGCGTCCTGTTCA	AB_187219.1	[28]
	R	GGTGACCCTGACCGATGT		
Nuclear oligomerization domain 2 (NOD2)	F	GAGCGCATCCTCTTAACTTTC	AB426547.1	[28]
	R	ACGCTCGTGATCCGTGAAC		
Proliferating cell nuclear antigen (PCNA)	F	GTTGATAAAGAGGAGGAAGCAGTT	NM_001291925.1	[28]
	R	TGGCTTTTGTAAAGAAGTTCAGGTAC		
Peptidylprolyl isomerase A (cyclophilin A)	F	CCCACCGTCTTCTTCGACAT	NM_214353.1	[38]
	R	TCTGCTGTCTTTGGAACTTTGTCT		
Prion protein (PRP)	F	TTTGTGCATGACTGCGTCAAC	NM_001008687.1	Present study
	R	CGTGGTCACTGTGTGCTGCT		
Ribosomal protein L32 (RPL32)	F	AGTTCATCCGGCACCAGTCA	NM_001001636.1	[26]
	R	GAACCTTCTCCGCACCCTGT		
Suppressor of Cytokine Signaling 3 (SOCS3)	F	CTTCACGCTCAGCGTCAAG	HM045422.1	Present study
	R	CTTGAGCACGCAGTCGAAG		
Transforming growth factor beta (TGFB)	F	GAAGCGCATCGAGGCCATTC	NM_214015	[28]
	R	GGCTCCGGTTCGACACTTTC		
Toll-like receptor 1 (TLR1)	F	TGCTGGATGCTAACGGATGTC	AB219564.1	[28]
	R	AAGTGGTTTCAATGTTGTTCAAAGTC		
Toll-like receptor 2 (TLR2)	F	TCACTTGTCTAACTTATCATCCTCTTG	AB085935.1	[28]
	R	TCAGCGAAGGTGTCATTATTGC		
Toll-like receptor 4 (TLR4)	F	GCCATCGCTGCTAACATCATC	AB188301.2	[28]
	R	CTCATACTCAAAGATACACCATCGG		
Toll-like receptor 5 (TLR5)	F	CCTTCCTGCTTCTTTGATGG	NM_001348771	[28]
	R	CTGTGACCGTCCTGATGTAG		
Toll-like receptor 6 (TLR6)	F	AACCTACTGTCATAAGCCTTCATTC	AB085936.1	[28]
	R	GTCTACCACAAATTCACTTTCTTCAG		
Tumor Necrosis Factor alpha (TNF-A)	F	ACTGCACTTCGAGGTTATCGG	NM_214022	[28]
	R	GGCGACGGGCTTATCTGA		
Zonula occludens 1 (ZO-1)	F	ATAACATCAGCACAGTGCCTAAAGC	AJ318101.1	Present study
	R	GTTGCTGTTAAACACGCCTCG		
